# KinaseMD: kinase mutations and drug response database

**DOI:** 10.1093/nar/gkaa945

**Published:** 2020-11-02

**Authors:** Ruifeng Hu, Haodong Xu, Peilin Jia, Zhongming Zhao

**Affiliations:** Center for Precision Health, School of Biomedical Informatics, The University of Texas Health Science Center at Houston, Houston TX 77030, USA; Center for Precision Health, School of Biomedical Informatics, The University of Texas Health Science Center at Houston, Houston TX 77030, USA; Center for Precision Health, School of Biomedical Informatics, The University of Texas Health Science Center at Houston, Houston TX 77030, USA; Center for Precision Health, School of Biomedical Informatics, The University of Texas Health Science Center at Houston, Houston TX 77030, USA; Human Genetics Center, School of Public Health, The University of Texas Health Science Center at Houston, Houston TX 77030, USA; MD Anderson Cancer Center UTHealth Graduate School of Biomedical Sciences, Houston TX 77030, USA

## Abstract

Mutations in kinases are abundant and critical to study signaling pathways and regulatory roles in human disease, especially in cancer. Somatic mutations in kinase genes can affect drug treatment, both sensitivity and resistance, to clinically used kinase inhibitors. Here, we present a newly constructed database, KinaseMD (**kinase m**utations and **d**rug response), to structurally and functionally annotate kinase mutations. KinaseMD integrates 679 374 somatic mutations, 251 522 network-rewiring events, and 390 460 drug response records curated from various sources for 547 kinases. We uniquely annotate the mutations and kinase inhibitor response in four types of protein substructures (gatekeeper, A-loop, G-loop and αC-helix) that are linked to kinase inhibitor resistance in literature. In addition, we annotate functional mutations that may rewire kinase regulatory network and report four phosphorylation signals (gain, loss, up-regulation and down-regulation). Overall, KinaseMD provides the most updated information on mutations, unique annotations of drug response especially drug resistance and functional sites of kinases. KinaseMD is accessible at https://bioinfo.uth.edu/kmd/, having functions for searching, browsing and downloading data. To our knowledge, there has been no systematic annotation of these structural mutations linking to kinase inhibitor response. In summary, KinaseMD is a centralized database for kinase mutations and drug response.

## INTRODUCTION

Protein kinases (PKs) represent one of the largest recognized protein groups that are involved in multiple biological processes. More than 30% of all human proteins can be modified by PK activities (1). In addition, PKs are the enzymes for the process of phosphorylation, which play critical roles in the regulation of almost all biological processes and pathways in eukaryotes (2). Therefore, dysfunction of PKs and their downstream substrates has been involved in various human diseases, especially in cancer (3,4). To molecularly target the activated kinases in cancer patients, >250 kinase inhibitors (KIs) are currently undergoing clinical trials and 48 have been approved for patient treatment by Food and Drug Administration (FDA), such as imatinib, gefitinib, sorafinib, erlotinib, dasatinib and crizotinib (5,6). However, studies have shown that many somatic point mutations affect drug treatment or even induce drug resistance to the commonly used KIs (7,8). Accordingly, the identification of actionable mutations in PKs has long been a hot topic, which contributes to molecularly targeted therapies for better precision medicine. There is growing evidence that KI-resistant mutations mainly fall into four kinase domain regions: gatekeeper, A-loop, G-loop and αC-helix (7,9–13). These four substructures are well studied for their roles in causing drug resistance due to secondary mutations (e.g. acquired mutations due to drug treatment in cancer). One notable example is the T790M mutation in the epidermal growth factor receptor (EGFR). T790M mutation of EGFR is a gatekeeper mutation, which is associated with resistance to EGFR tyrosine kinase inhibitors such as erlotinib (3,14). Moreover, PKs are the most studied proteins for the phosphorylation changes caused by mutations. Many unique sequence motifs have been reported to affect or rewire signaling pathways and networks (15–17). For instance, Liu *et al.* reported a cancer patient-derived mutation R81T on the SIN1 protein. R81T impairs phosphorylation of the protein, leading to the hyper-activation of mTOR Complex 2 (mTORC2) and facilitates tumorigenesis (18).

Due to the importance of PKs in both basic and translational research, several databases have been developed with specific aspects to these PKs. These databases can be divided into three categories based on their contents and purposes. The first category focuses on collections of kinases as well as their family and domain annotations. KinBase (19), KinG (20) and KinWeb (21) are three databases in this category. The second category focuses on annotations of kinase structures and/or KI data, e.g. KIDFamMap (22) and PKIDB (23). The third category focuses only on collections of kinase mutations, including KinMutBase (24) and MoKCa (25). However, most of these databases lack timely update and the contents stay behind the fast evolving research progress. More importantly, there has been no database available so far that integrates PK mutations and the related drug response, especially those mutations causing drug resistance due to the potential change of protein substructures. Furthermore, somatic mutations contribute to the development of cancer through the reconfiguration of phosphorylation signaling. Thus, the analysis of mutations with PK regulatory networks may reveal novel mechanisms (26,27).

Here, we introduced a newly constructed database called KinaseMD to annotate the latest PK mutations and also unique drug responses from multiple sources in >33 cancer types. In our database, we integrated 679 374 PK-related somatic mutation records from five cancer-related datasets [Cancer Cell Line Encyclopedia (CCLE) (28), Genomics of Drug Sensitivity in Cancer (GDSC) (29), The Cancer Genome Atlas (TCGA), International Cancer Genome Consortium (ICGC) (30) and Catalogue of Somatic Mutations in Cancer (COSMIC) (31)] and systematically investigated these mutations, especially those that fall in four types of kinase functional substructures that affect both drug sensitivity and resistance. These four substructures are gatekeeper, A-loop, G-loop and αC-helix. We detected mutation hotspots in those substructures that are associated with drug resistance. Both genome-wide annotations from curation and newly generated analysis results are integrated into KinaseMD for a better understanding of these special protein structures. Moreover, we investigated the influence of mutations on kinase-specific phosphorylation networks and identified >250 000 network-rewiring entries. The functional outcomes are annotated in KinaseMD and classified into four types: gain, loss, up-regulation and down-regulation. In short, KinaseMD deposits the latest human PKs with comprehensive annotations, including function descriptions, PK classifications, mutations identified in cancers, drug responses and functional network-rewiring events. KinaseMD provides a landscape view of drug resistance-associated mutation hotspots in functional kinome substructures, which is unique from other kinase databases. To better serve the community, KinaseMD website provides multiple functions for searching, filtering, browsing and visualization of the data.

## DATA COLLECTION AND PROCESSING

**Figure 1.**
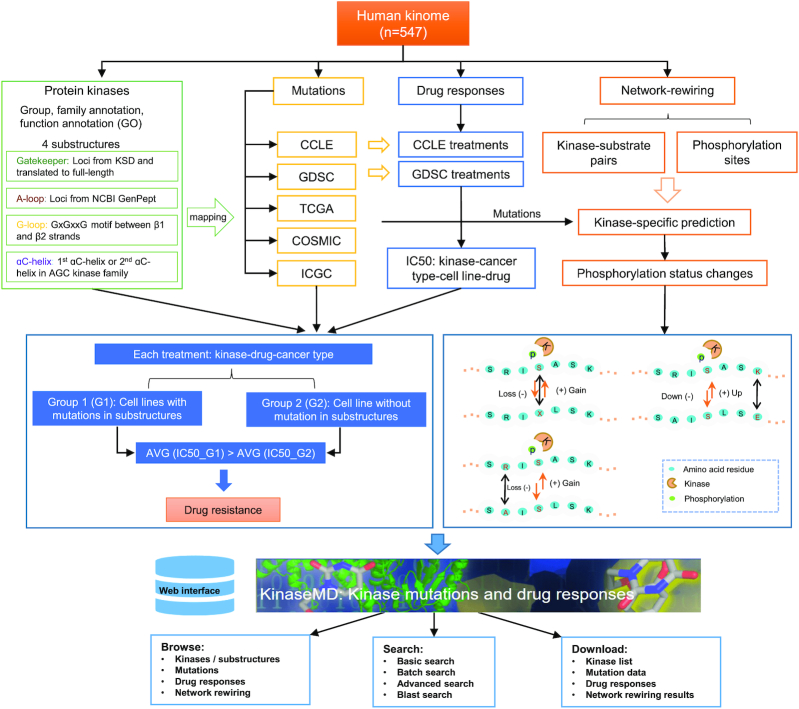
General pipeline for data collection, processing, curation, analysis, and website functions of the KinaseMD database.

Figure 1 summarizes the general pipeline for the data collection, processing, curation, analysis and website functions for the KinaseMD database.

### Collection of protein kinases

We first collected the human PKs from KinBase (19) (August 2019) and extended the list by combining the latest PK list from the UniProt Knowledgebase (32) (UniProtKB, February 2020). All the collected PKs were mapped and supported by the latest records in HGNC (33) (February 2020), UniProtKB (February 2020), Ensembl (34) (February 2020) and NCBI RefSeq (35) (February 2020) databases. The classification information of all PKs (group, family and subfamily) was curated from KinBase and UniProtKB and validated by PhosphoSitePlus (36). The protein sequences and domain information of PKs, including the domain names and start and end positions, were obtained from UniProtKB. Functional annotations were included such as Gene Ontology (GO) annotations. All 3D structure files (PDB files) of PKs were downloaded from the RCSB PDB (37) website through retrieve/ID mapping between UniProtKB and PDB database.

### Annotations of four types of kinase functional substructures

To identify regions of each of the four types of substructures in PKs, we collected the information from three resources: Kinase Sequence Database (38) (KSD, July 2016), the UniProtKB (February 2020) and the protein database within NCBI (February 2020).

#### Gatekeepers in human PKs

Gatekeeper residue in PKs is a single amino acid located near the protein-drug binding site. Gatekeeper mutation changes the residue from a small side chain, which sterically accommodates drugs, to a bulky side chain, which impedes drug-protein binding, and thus, it introduces potential drug resistance. The locus information for gatekeeper was mainly collected from KSD. There is gatekeeper information in KSD for 946 kinase domain sequences. We aligned these domain sequences to the full-length sequences of PKs by using the Basic Local Alignment Search Tool for Protein (BLASTP) (39,40). Only the alignment results with 100% identity hits were retained.

#### A-loops in human PKs

A-loop is also called the activation loop or T-loop that is located in the C-lobe of a protein. There is a phosphorylation site within the A-loop region, which can induce a conformational change of the loop to allow substrate binding (41). A-loop mutations may increase entropy or destabilize the inactive conformation that will disfavor the drug binding (42,43). In the collection of A-loop regions in each kinase protein sequence, we only selected those kinases that are marked as ‘Reviewed’ and have domain locus information in UniProtKB. Then, we searched the GenPept from NCBI Protein using batch Entrez for the A-loop information.

#### G-loops in human PKs

G-loop is also named P-loop (phosphorylation loop) and glycine-rich loop. The typical sequence motif of G-loop is GxGxxG, where G represents glycine, and x can be any amino acid (11). Mutations in G-loop regions can lead to destabilization of the inactive conformation and stabilization of the active conformation. Therefore, it will remove direct drug interactions and cause clinical resistance to type 2 kinase inhibitors. In literature, the G-loop lies between the β1 and β2 strands and contains a consensus GxGxxG motif (44,45). The gff file was downloaded from UniProtKB that describes the β strand regions for all human PKs. We first navigated the locations of the first two β strands (β1 and β2) within the kinase domain for each PK. In our search process, we applied a window of ± 2 amino acids (AAs) because some kinases have their first β strand location before the starting point of the kinase domain. Then, for the PKs that have at least two β strands in their kinase domain, we searched for the GxGxxG motif between the β1 and β2 strands in the kinase domain. If there is no complete secondary structure information for a PK in UniProtKB, the first 22 residues in the kinase domain were checked for the GxGxxG motif with ± 2 AA window, as we found that the G-loop motif usually occurred in the first 20 amino acids in the kinase domain. We searched all the kinase domains separately if a PK had more than one kinase domain.

#### αC-helix in human PKs

The αC-helix is a single α-helix located in the N-lobe of the kinase domain between β3 and β4 strands. αC-helix is usually the first α-helix in the kinase domain (from N-terminus), but in some kinases such as AGC kinases (the PKA, PKG and PKC groups, see ‘database content and usage’ section), a short αB–helix may precede αC-helix (46). To locate the positions of αC-helix, we applied a similar search strategy as we used to search for G-loop regions. Briefly, we first confirmed the range of the kinase domains of each PK and determined all the α-helix regions inside these domains based on the full-length protein sequences. Next, the first α-helix was chosen as the αC-helix except for AGC kinases. Because the kinase domain usually starts with a β strand, we did not check if the first α-helix occurs outside the kinase.

### Mapping point somatic mutations in cancer to protein substructures

Somatic mutations could change kinase conformations and subsequently affect drug bindings or the transition between active and inactive status. Somatic mutation data were downloaded from five cancer-related datasets: CCLE, GDSC, TCGA, GDSC and COSMIC. We only kept the nonsynonymous mutations, because synonymous mutations do not change the protein sequences. All the AA mutations were mapped to protein sequences. The mutations were kept when the reference residue at the mutation point matched the one at the full-length amino acid sequence. Then, the somatic mutations were mapped to substructure regions of human PKs for pan-cancer analysis. If a mutation position occurred in multiple samples, we saved all the identifiers of those samples defined by each dataset in the results.

### Drug response data associated with PKs and substructures

We hypothesized that mutations in the four kinds of substructure regions could change the conformation of kinase structure and impact drug binding improperly. The CCLE project conducted a genetic and pharmacologic characterization of a large panel of human cancer cell lines and provided public access to the drug response data of 24 drugs over 1000 cell lines. The GDSC project tested drug response by treating >1000 cancer cell lines that have somatic mutation profiles with 265 anti-cancer drugs. Both projects included kinase inhibitors. To systematically investigate the candidate mutations associated with drug response in cancer, raw drug response data associated with human kinases were collected from CCLE and GDSC, along with the cell line annotation files (CCLE cell lines, GDSC cell lines). Only the drug treatments that targeted the kinase proteins were included for downstream analysis.

To explore the drug resistance-associated mutations, we first stratified all CCLE and GDSC cell lines by cancer type. For each cancer type, we divided the cell lines into two groups for each drug following the two rules: (i) group 1 (G1) included the cell lines with mutations in the substructures and group 2 (G2) included the cell lines without mutations in any of the four substructure regions or (ii) G1 included the cell lines with a primary mutation in the substructures and G2 included the cell lines possessing a secondary mutation besides the primary mutation in any of the four substructure regions. We defined a treatment as a drug-kinase-cancer type combination and conducted this analysis separately for each treatment. We defined drug resistance if the average IC50 value of G1 was larger than G2.

### Impact of mutations on kinase-specific phosphorylation network

We investigated the AA substitutions for their potential impact on protein phosphorylation as well as phosphorylation signaling networks. Following the definition of previous work (47), we categorized our curated AA changes into three groups: those that directly change a phosphorylation site (*p*-site), those that are located proximal to the nearest phosphorylation site (1–2 AAs) and those that are located distal to the nearest phosphorylation site (3–7 AAs). To estimate the network impact of each AA change, we performed phosphorylation status analysis using the kinase-specific predictor of GPS 5.0 software (48). In this work, we analyzed AA changes in **s**ite-**s**pecific **k**inase–**s**ubstrate **i**nteractions (ssKSIs) for each of our curated AA substitutions. By comparing the phosphorylation status between the reference sequence (Score1) and the sequence harboring the point mutation (Score2), all candidate network-rewiring mutations were classified into four categories based on the predefined cutoffs in the predictor (Figure 1): (i) gain, a new ssKSI in kinase–substrate pair was gained (i.e. Score2 > cutoff > Score1); (ii) loss, an existing ssKSI in a kinase–substrate pair was lost (i.e. Score2 < cutoff < Score1); (iii) up-regulation, the level of the ssKSI was increased (i.e. Score2 > Score1 > cutoff); and (iv) down-regulation, the level of the ssKSI was decreased (i.e. Score1 > Score2 > cutoff).

## DATABASE CONTENT AND USAGE

KinaseMD hosts the latest human PKs with comprehensive annotation data for structural mutations, functional mutations and drug responses in pan-cancers. Table 1 summarizes the data in KinaseMD. A user-friendly website is developed to display the curated data and analysis results and provide functions for searching, filtering, browsing and visualization of the data.

**Table 1.** Summary of datasets in KinaseMD

Dataset	# kinases	Group (# kinases)		Others
Human protein kinases	547	TK (90), TKL (43), AGC (64), CAMK (75), CMGC (62), CK1 (12), RGC (5), STE (47), Atypical kinases (48), Others (101)		1485 domain records, 6249 PDB files
Kinase with substructures	388	Gatekeeper (344), A-loop (312), G-loop (172), αC-helix (231)		
	**# counts**	**Source dataset (# counts)**		
Mutation records	679 374	COSMIC (392 345), ICGC (162 557), TCGA (66 528), CCLE (34 096), GDSC (23 848)		
Mutation positions	180 856	COSMIC (139 333), ICGC (114 609), TCGA (53 050), CCLE (28 372), GDSC (19 100)		
Mutations in substructures	32 997	A-loop (23 864), G-loop (3625), αC-helix (3415), Gatekeeper (2093)		
Mutation positions in substructures	4742	A-loop (3042), G-loop (634), αC-helix (967), Gatekeeper (111)		
	**# counts**	**# drugs**	**# cell lines**	**# kinases**
Drug response records	390 460	204	1489	129
Drug resistance records	137	80	77	41
	**# kinases**	**# substrates**	**# kinase–substrate interactions**	**# events**
Network-rewiring effects	296	2647	6636	251 522
	**# gains**	**# losses**	**# up-regulations**	**# down-regulations**
	15 205	43 448	86 861	106 008

### Data summary

#### PK classification

In the current version of KinaseMD, there were 547 human PKs with the group, family and subfamily information. These PKs were divided into 10 groups following the classification scheme from KinBase: (i) tyrosine kinase group (TK, *n* = 90); (ii) tyrosine kinase-like group (TKL, *n* = 43); (iii) the PKA, PKG and PKC group (AGC, *n* = 64); (iv) calcium/calmodulin-dependent protein kinase (CAMK, *n* = 75); (v) the CDK, MAPK, GSK3, CLK families (CMGC, *n* = 62); (vi) casein kinase 1 group (CK1, *n* = 12); (vii) receptor guanylate cyclase (RGC, *n* = 5); (viii) the MAP kinase cascade kinases, homologs of yeast Ste7, Ste11 and Ste20 kinases (STE, *n* = 47); (ix) atypical kinases (*n* = 48); (x) others (*n* = 101). Basic functional annotations were curated for all PKs and the detailed data were available in Supplementary Table S1. In total, 1485 domain records and 6249 PDB files were obtained and made available for visualization in KinaseMD.

#### Four types of substructures in PKs

**Figure 2.**
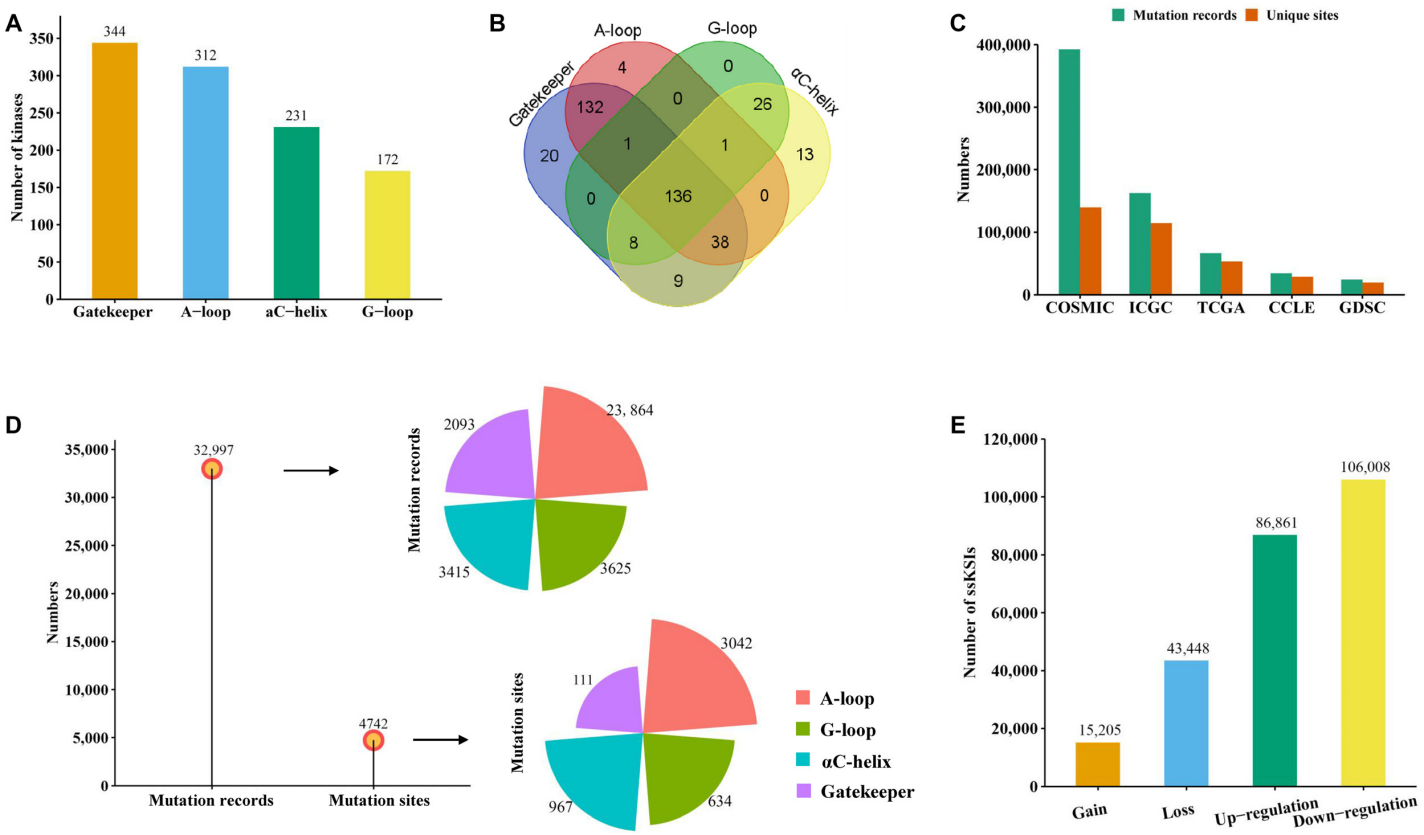
Statistics of kinases, substructures, mutations and network rewiring information. (**A**) Number of PKs in each of the four substructures. (**B**) Venn diagram shows the overlapped number of kinases among the four substructures. (**C**) The number of collected nonsynonymous somatic mutations from five cancer-related datasets and the number of unique mutation sites in each dataset. (**D**) The number of mutated samples and mutated sites in four types of substructures in human PKs. (**E**) Summary of network-rewiring outcomes based on nonsynonymous mutations: gain, loss, up-regulation and down-regulation; ssKSI, site-specific kinase–substrate interactions.

We searched the information on substructure locus of the 547 human PKs from KSD, UniProtKB and NCBI protein databases. We first filtered out the PKs without locus information in any of the four substructures. As a result, we found 388 unique human PKs having at least one type of functional substructure. More specifically, there were 344, 312, 231 and 172 kinases that had gatekeeper, A-loop, αC-helix and G-loop substructures, respectively (Figure 2A). Among these PKs, 37 had only one substructure, while 136 had all four substructures. The detailed information is summarized in Supplementary Table S2 and Figure 2B.

#### Kinase and substructure mutations

After filtering out synonymous mutations and removing the unmatched records, we had ∼3.9 million somatic mutation records from the five cancer-related datasets and further obtained 679 374 somatic mutations associated with human PKs assigning on 180 856 unique AA positions (abbreviated as AAps) (Figure 2C, COSMIC: 392 345 AAps, ICGC: 162 557 AAps, TCGA: 66 528 AAps, CCLE: 34 096 AAps, GDSC: 23 848 AAps, Table 1). These mutations were then mapped to the regions of the four substructures of the 388 kinases. Finally, 32 997 mutation records on 4742 unique AAps were obtained (Figure 2D, A-loop: 23 864 mutation records/3042 AAps, G-loop: 3625 mutation records/634 AAps, αC-helix: 3415 mutation records/967 AAps, gatekeeper: 2093 mutation records/111 AAps, Table 1). For each substructure, the mutated AA positions with most mutation records (top 3) are summarized here. Gatekeeper: EGFR T790 (1397 mutation records), ABL1 T315 (329 mutation records) and KIT T670 (39 mutation records). A-loop: EGFR L858 (10 455 mutation records), KIT D816 (2154 mutation records) and PDGFRA D842 (710 mutation records). G-loop: EGFR G719 (724 mutation records), BRAF G469 (317 mutation records) and ABL1 E255 (161 mutation records). αC-helix: KIT K642 (178 mutation records), ABL1 G250 (137 mutation records) and GRK4 R222 (57 mutation records). Supplementary Table S3 provides detailed data about the substructure mutations.

#### Drug response and drug resistance

We collected 390 460 drug response records associated with human PKs from CCLE and GDSC projects. By comparing the average IC50 values of each treatment on the two groups of cell lines (Supplementary Table S4), 137 treatments were identified to be potential drug resistance events (Supplementary Table S5). As one example, in LUAD cell lines treated with Afatinib, the cell lines harboring only the L858 mutation had an average IC50 of 0.02 μM, whereas the cell lines with both T790 and L858 mutations had an average IC50 of 0.96 μM. Here, T790 is a gatekeeper AA mutation in EGFR.

#### Mutation network-rewiring effect

More than 530 000 experimentally identified *p*-sites on 30 637 human proteins were obtained from the Eukaryotic Phosphorylation Site Database (EPSD) (49). Moreover, 11 776 high-quality (experimentally determined only) kinase–substrate interactions (KSI) were acquired from PhosphoSitePlus (36). Overall, we compiled 6636 unique KSI pairs connecting 296 kinases and 2647 nonkinase substrate proteins. Based on the datasets, we explored the influence of mutations on kinase-specific *p*-sites. We identified >250 000 ssKSIs records, including 15 205 gain, 43 448 loss, 86 861 up-regulation and 106 008 down-regulation ssKSIs, respectively (Figure 2E, the full list of kinase regulatory network rewiring events caused by mutations can be downloaded from KinaseMD website: https://bioinfo.uth.edu/kmd/download.html).

### Web design and interface

The web interface of KinaseMD was implemented using PHP and Bootstrap 4 (http://getbootstrap.com/). All the processed data, annotation information and summary statistics were stored in the MySQL database system. The dynamic web pages were implemented by utilizing several JavaScript libraries and Ajax strategies. The visualization of interactive charts was created by using highcharts (https://www.highcharts.com/). We provided five modes for users to easily browse the data and four options to search the database. A tutorial page is available at https://bioinfo.uth.edu/kmd/tutorial.html.

#### Browse

Users can browse all the curated and processed data through the Browse function. The available options include the human PKs (including the substructure regions), all mutations across the PKs, the drug response data associated with PKs, the network-rewiring events caused by mutations and the potential drug resistance results in mutation hotspots. The quick-access buttons were provided on the home page for checking these datasets. We provided both a tree view and a data table view to present PK classifications and the basic information for each PK. In the tree view, users can select the group, family and subfamily names to display the detailed annotation information for the corresponding category. Users can also search corresponding records by typing keywords on the top right of the table in each browsing interface, while links were added in the last column to a detailed information page of each PK.

#### Search

KinaseMD provides four search options, including general search, batch search, advanced search and BLASTP search. Users can conveniently query the database with one or multiple keywords. For the general search, users can directly search the KinaseMD database by selecting a keyword type following by a specific keyword. For example, if the keyword ‘CDK2’ of ‘Gene symbol’ is submitted, the corresponding results are shown in a tabular format, including UniProt ID, gene symbol, gene names, PK classification, chromosome and more details. Detailed annotations can be retrieved through the ‘More’ link. In batch search, users can enter multiple keywords, such as gene ID, UniProt ID, gene symbol or PK classifications in a line-by-line format for querying. For example, users can submit multiple UniProt IDs as keywords, such as ‘P24941’, ‘P15056’, ‘P53350’, ‘P31749’ and ‘P00519’. In advanced search, users could use relatively complex and combined keywords to locate precise information, with up to three search terms. The interface of the search engine allows querying by different database fields and the linking of queries through three operators ‘and’ and ‘or’. The option of BLASTP search was designed for querying the KinaseDB by protein sequences. The blastp program of NCBI BLAST packages was included in the database. Users can enter a protein sequence in FASTA format to search identical or homologous proteins. For example, if the CDK2 protein sequence and a user-defined ‘*E*-value’ is submitted, the corresponding homologous proteins greater than that threshold will be listed in a result table.

#### PK annotation page

**Figure 3.**
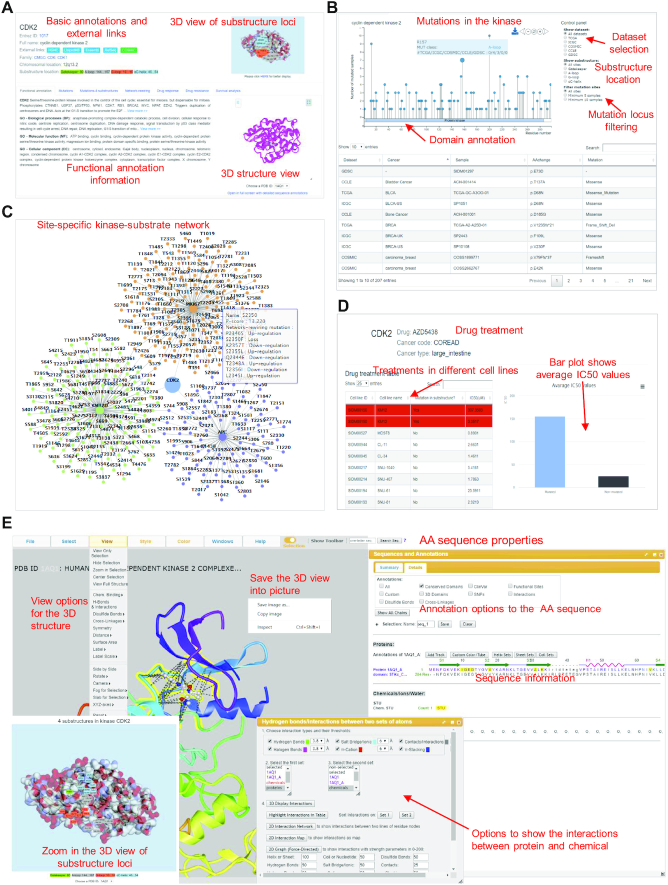
The kinase annotation pages in the KinaseMD database. (**A**) The basic annotation information for the kinase proteins. A 3D structure view is provided for the user to look into the amino acid sequence character. (**B**) The ‘Mutation’ tab shows detailed mutations. The needle plot on the top displays the sample counts for each AA position. By choosing the option on the right corner, the plot will be displayed dynamically. A table at the bottom shows the full list of mutations in the selected kinase in the samples from the five cancer-related datasets. (**C**) The network rewiring tab shows the site-specific kinase regulatory network affected by the mutations. (**D**) The drug resistance tab shows the drug treatment information and how drug resistance is calculated from drug response data. (**E**) 3D protein structure view page provides options for users to interact with the AA sequence.

The PK page (Figure 3) starts with basic annotation information, including the gene symbol and name, Entrez ID linked to NCBI, multiple cross-referencing (HGNC, UniProtKB, Ensembl, Refseq and COSMIC), PK classification, chromosomal location, substructure region, and an interactive 3D view of substructure region at the top part of the page. This page presents six tabs to show the detailed annotations. (i) Basic functional annotations. It contains the functional description of selected PK and the relevant GO terms. The 3D structural view is provided for the user to look into the AA sequence and its ligand characters. (ii) Mutations. The mutation data are shown for the selected PK in this tab. The needle plot on the top displays the sample counts at each mutation position. The control panel on the right side is allowed to choose a specific mutation dataset (e.g. mutation source, mutations in substructure regions and filtering mutations based on the number of mutated samples) and the plot will change dynamically. A table at the bottom shows the full list of mutations for the selected PK in different samples from the five cancer-related datasets. (iii) Mutation in substructure regions. In this tab, mutations in the four types of substructure were listed. A bar plot displayed the number of mutated samples in each substructure. After clicking on the bar, it will show the number of mutated samples from each cancer dataset. (iv) Network rewiring. It shows kinase regulatory network affected by mutations. The number of functional mutations is summarized and shown in a bar plot. It gives users an intuitional view of the main consequences that may be caused by mutations in this PK. A detailed table is provided to check the consequence from a specific mutation. (v) Drug response. All drug treatment data associated with the selected PK are listed for different cancer types in multiple cell lines. (vi) Drug resistance. If potential drug resistance is found for the selected PK having mutations in the four substructures, corresponding drugs and cancer types will be displayed in this tab. By clicking the ‘more’ link, it will direct users to a detailed page showing how the drug resistance is calculated from drug response data.

#### Download of data, figures and tables

All the plots (e.g. needle plot, bar plot and network plot) generated in the database can be downloaded by clicking the ‘Download’ button with multiple formats. On the ‘Download’ page of the KinaseMD website, we provide the download links for users to fully access our processed data.

## DISCUSSION AND CONCLUSION

Both activated and mutated PKs are related to the occurrence and development of cancer. A good number of kinase inhibitors targeting the activated kinase have been approved for patient treatment. Accordingly, annotating and studying structural and functional mutations in human PKs will be important for better understanding the mechanisms of cancer development and drug treatment in the era of precision medicine. KinaseMD is a newly constructed database that systematically annotates PK-related mutations implicated in functional protein substructures, rewiring of phosphorylation signaling and drug response. We uniquely collected and curated four types of protein substructures, i.e., gatekeeper, A-loop, G-loop and αC-helix, which are reported to cause drug resistance due to secondary mutations in human PKs. Such database has not been developed before. We curated mutation data mapped to these substructures in 547 human PKs through mining large-scale cancer data sets (TCGA, ICGC, COSMIC, CCLE and GDSC) and protein structure information from various sources. We also report drug resistance mutation hotspots in those substructures. We further annotated functional mutations that might alter the PK regulatory network. We compiled a large number of experimentally identified *p*-sites and KSI pairs and evaluated the mutations causing the change in the ssKSI status. We assigned four functional outcome measures: gain, loss, up-regulation and down-regulation.

Moreover, cancer is the outcome (e.g. abnormal cell growth and migration) of the acquisition of somatic mutations. A cancer genome can harbor tens to thousands of somatic mutations, but only a few are driver mutations. Critical mutations in cancer genomes have been demonstrated with their strong impact on the clinical effectiveness of drug treatment, leading to highly variable drug response. Due to the complexity of mutations and biology in cancer cell lines, it is difficult to determine the one specific mutation that is the only factor affecting drug response. This complexity complicates the linking of specific mutations to drug response or changes in the phosphorylation network. KinaseMD provides data resources and features for the research communities to investigate the specific contribution of a mutation to the drug response or functional network changes.

All the data in our database were collected from published studies or calculated based on accepted theories. As evidenced in literature and knowledgebases, mutations can impact the drug response during cancer treatments, both sensitivity and potential resistance. To bridge the gap between mutations and drug response, we hypothesized that mutations in the four kinds of substructure regions could change the conformation of kinase structure and impact drug binding improperly. As in literature, mutations in these four kinds of substructure regions impact protein conformation changes (50–52), and these changes can result in drug response variation (53) and kinase dysfunction (54). As this hypothesis has been supported by previous publications, we applied it to all the protein kinases and collected drug response data.

In summary, KinaseMD is a unique database that provides the most updated data on mutations, drug response and functional sites of PKs. It will become a valuable resource for further study of biological, structural and translational aspects of kinases. We will regularly update the KinaseMD and incorporate the future released data.

## DATA AVAILABILITY

All data are available from the KinaseMD (https://bioinfo.uth.edu/kmd/).

## Supplementary Material

gkaa945_Supplemental_Files
