# Supplementary material for: KinaseMD: kinase mutations and drug response database
Source: Nucleic Acids Res. 2020 Nov 2;49(D1):D552–61. doi: 10.1093/nar/gkaa945 (PMC7779064; doi:10.1093/nar/gkaa945)
Supplement: gkaa945_Supplemental_Files [file gkaa945_supplemental_files.zip › KinaseMD Supplementary.docx]

**KinaseMD Supplementary Files**

Supplementary Table S1. Full list of human protein kinases in KinaseMD.

Supplementary Table S2. List of the substructure regions.

Supplementary Table S3. List of all mutations in regions of substructures.

Supplementary Table S4. List of drug responses and substructure mutations in cell lines.

Supplementary Table S5. List of potential drug resistance in kinase inhibitor treatments.
